# Hyaluronan, fluid pressure, and stromal resistance in pancreas cancer

**DOI:** 10.1038/bjc.2012.569

**Published:** 2013-01-08

**Authors:** P P Provenzano, S R Hingorani

**Affiliations:** 1 Clinical Research Division, Fred Hutchinson Cancer Research Center, Seattle, WA 98109, USA; 2 Public Health Sciences Division, Fred Hutchinson Cancer Research Center, Seattle, WA 98109, USA; 3 Division of Medical Oncology, University of Washington School of Medicine, Seattle, WA 98195, USA

**Keywords:** pancreatic ductal adenocarcinoma, hyaluronic acid, interstitial fluid pressure, stromal resistance, desmoplasia, hyaluronidase

## Abstract

Pancreatic ductal adenocarcinomas (PDAs) are notoriously aggressive and resistant to treatment. They distinguish themselves further by their robust fibroinflammatory, or desmoplastic, stromal reaction and degree of hypovascularity. Recent findings have revealed multiple mechanisms of stromal complicity in disease pathogenesis and resistance. In this review, we focus on altered physicomechanics as one mechanism of what we term as ‘stromal resistance’ in PDA. Extremely high interstitial fluid pressures and a dense extracellular matrix combine to limit the delivery and distribution of therapeutic agents. We discuss the genesis and consequences of altered fluid dynamics in PDA and strategies to restore them.

Pancreatic ductal adenocarcinoma (PDA) is an aggressive disease of the exocrine pancreas, marked by a 5-year survival rate of <5% and a median survival of ∼6 months, making it the fourth leading cause of cancer-related deaths in the United States (Hidalgo, 2010; Siegel *et al*, 2012). The anatomy and biology of PDA conspire to elude detection and resist eradication. An absence of localising symptoms, together with a lack of biomarkers and screening methods for early detection, irreparably delay diagnosis. Indeed, 80–90% of patients are diagnosed too late to permit surgical intervention, which itself can prolong life but is seldom curative, due to almost inevitable disease recurrence and/or metastasis (Allison *et al*, 1998; Oettle *et al*, 2007). For these patients, treatment is confined to chemical and/or radiotherapies, which historically have provided limited benefit at best.

The deoxycytidine analogue, gemcitabine, has represented the standard-of-care for disseminated PDA, although this may be slowly changing. Gemcitabine monotherapy can improve quality of life in a subset of patients and extend survival modestly (Burris *et al*, 1997). The poor response of PDA to chemotherapy was long thought to result principally from cellular mechanisms of intrinsic resistance in the tumour epithelium; it is now understood, however, to be due at least in part to an inability of even small molecule therapeutics to enter and perfuse the tumour bed (Olive *et al*, 2009). Intriguingly, sustained exposure of PDA to chemotherapies with long circulating half-lives can increase intratumoral drug levels and improve response. For example, nab-paclitaxel, an albumin-coated nanoparticle formulation of taxol with a >10 h half-life, in combination with gemcitabine improved median survival (mOS) substantially compared with historical controls (mOS=12.2 months; Von Hoff *et al*, 2011). In a large phase III trial of patients with metastatic disease, the multi-drug regimen FOLFIRINOX – which combines bolus doses of oxaliplatin, irinotecan, leucovorin, and 5-fluorouracil, followed by a prolonged 46h continuous infusion of fluorouracil – improved survival compared with gemcitabine alone (mOS=11.1 versus 6.8 months, respectively; Conroy *et al*, 2011). An admittedly substantial side-effect profile may limit widespread use of FOLFIRINOX and, despite representing potentially significant advances over the prior collective experience, neither regimen is curative.

Our expanding knowledge of the genetic repertoire of pancreas cancers promises to guide development of new, more selective strategies (Iacobuzio-Donahue, 2012). The basic molecular profile of PDA is known (Hruban *et al*, 2000). Activating mutations in the *KRAS* proto-oncogene occur early in disease progression and are found in >90% of invasive PDAs. Subsequent mutations in the *CDKN2A*, *TP53*, and/or *DPC4* (*SMAD4*) tumour suppressor genes are also common (>90%, ∼75%, and ∼50% respectively; reviewed in Maitra and Hruban, 2008). Genetic engineering in mice has demonstrated that these mutations, and the order in which they arise, drive, and shape disease progression (Aguirre *et al*, 2003; Hingorani *et al*, 2003, 2005; Izeradjene *et al*, 2007), although efforts to target them directly to treat pancreas cancer have yet to achieve fruition. A number of signalling pathways are also aberrantly regulated in PDA including *EGFR, ERBB2*, *COX-2*, *SHH*, *MMPs*, and *NOTCH*, and which suggest additional potential targets. To date, only inhibition of the epidermal growth factor receptor (EGFR) has been clinically approved, and even then provides only incremental improvement in survival (Moore *et al*, 2007).

In this review, we discuss the concept of ‘stromal resistance’ in PDA, namely the barriers to effective treatment imposed by the complex and dynamic tumour microenvironment (TME). In particular, we highlight physical mechanisms of drug resistance in PDA and recent efforts to target stromal elements in order to improve delivery of small molecule therapeutics. We discuss further how studies in genetically engineered mouse (GEM) models of PDA have yielded unexpected insights compared with more traditional xenograft and allograft models and may help to explain the previous divide between preclinical promise and clinical reality.

## Determinants of stromal resistance in PDA

**Figure 1:**
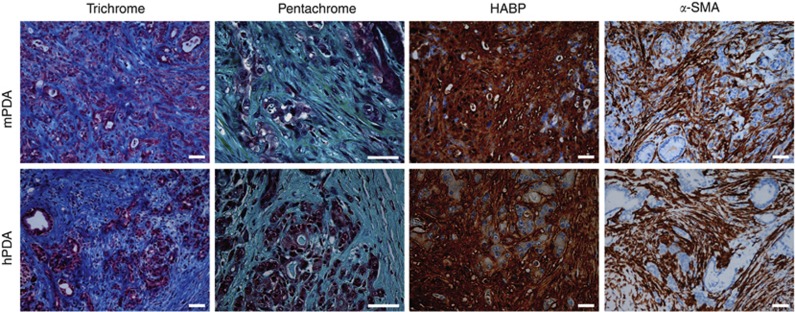
**The desmoplastic stroma in PDA.** Both mouse (top) and human (bottom) PDA display robust deposition of ECM and activated pancreatic stellate cells. Masson’s trichrome reveals robust collagen content in PDA (blue) while a more complex Movat’s pentachrome staining highlights the presence of GAGs and mucins (blue) co-localised with collagen (turquoise/green). Histochemistry with hyaluronic acid binding protein (HABP) confirms the abundance of HA in PDA and immunohistochemistry for *α*-SMA identifies activated PSC, or myofibroblasts. Scale bars=50 *μ*m.

A dynamic fibroinflammatory, or desmoplastic, response is a hallmark of PDA and essentially pathognomonic for the disease. This desmoplasia evolves during disease progression and includes stromal fibroblasts, immune cells, and excessive deposition of a complex extracellular matrix (ECM) (Figure 1; Mahadevan and Von Hoff, 2007). Activated stromal fibroblasts, or myofibroblasts, produce matrix constituents that alter the physical structure of the developing cancer ‘organ’ and also modulate tumour cell behaviour through direct binding interactions with surface receptors (Vonlaufen *et al*, 2008). The immune reaction in PDA consists largely of immunosuppressive and pro-tumourigenic elements that infiltrate at the earliest stages of preinvasive disease, with scant evidence of effector immunity (Clark *et al*, 2007). The ECM of this desmoplastic response contains significant levels of fibrous collagen together with a complex mixture of proteoglycans and glycosaminoglycans. In addition to roles in maintaining tissue integrity and providing signalling scaffolds, dense collagen and microfibrillary matrices and fluid-trapping mucopolysaccharides have been shown to impede diffusion (exchange driven by concentration gradients) and convection (pressure-driven fluid flow that drags molecules) through porous materials such as the stromal compartment in carcinomas (De Smedt *et al*, 1994; Brekken *et al*, 2000; Ramanujan *et al*, 2002). An elevated IFP that limits convection and a fibrotic ECM that compromises diffusion combine to impede solute flux across a perfused vessel and through a tumour bed (Fukumura and Jain, 2007; Tredan *et al*, 2007). Despite the presence of such substantial barriers to treatment, the altered fluid mechanics of the desmoplastic stroma in PDA represents an underappreciated and relatively unexplored mechanism of disease resistance.

## Hypovascularity and vascular collapse in PDA

The delivery and distribution of small molecules across a tissue are governed by well-defined principles of solute transport involving passive diffusive and convective fluxes (reviewed in Ogston and Michel, 1978). In general, the distribution of small molecules is primarily diffusion limited, while larger molecules rely also, or even preferentially, on convective flow. Diffusion is driven by concentration gradients between the intravascular and interstitial compartments, and convection by pressure gradients composed of hydrostatic and oncotic components. The balance between hydrostatic and oncotic pressures in tissues and vessels determines the net flow of fluid.

In contradistinction to most solid tumours, PDAs are hypoperfused; indeed, in standard clinical imaging modalities, they are identified as regions that take up less injected contrast material than the normal surrounding tissues (Park *et al*, 2009). Neuroendocrine tumours of the pancreas, on the other hand, appear bright on contrast-enhanced CT or MRI, reflecting their hypervascular nature. These radiographic signatures of blood flow provide tell-tale clues distinguishing these dramatically different tumour types. Perhaps not surprisingly, therefore, and despite a profound hypoxia (Koong *et al*, 2000) and elevated levels of pro-angiogenic factors (Buchler *et al*, 2003), PDAs have a paucity of blood vessels. Even worse, the vast majority of existing vessels in PDA are not functional (Olive *et al*, 2009; Provenzano *et al*, 2012). As a result, relatively few vessels are effectively perfused with chemotherapy, and those that are then face further barriers to transvascular distribution of drug into the stroma imposed by the ECM. Inhibition of paracrine sonic hedgehog signalling (Shh) in a GEM model of PDA depleted stromal fibroblasts, stimulated angiogenesis, and produced a salutary increase in cytotoxic drug delivery (Olive *et al*, 2009). Although these changes in tumour perfusion were short-lived, they provide proof-of-principle of a primary mechanism of drug resistance in PDA. However, the lack of significant functional perfusion in PDA results not only from a sparse vasculature but also from a profound degree of vascular collapse. In fact, ∼75% of the vessels in PDA appear to be collapsed (Provenzano *et al*, 2012). The observations raise immediate questions regarding the metabolic regulation in PDA cells and also the mechanisms underlying this vascular collapse.

## IFP is extremely elevated in PDA

A collapsed vasculature suggests the presence of interstitial pressures exceeding the combined intravascular hydrostatic and oncotic pressures and elastic forces of the vascular wall. As noted, a large body of work in a number of experimental model systems has suggested that altered fluid mechanics can limit the efficacy of systemic therapies (reviewed in Tredan *et al*, 2007). Fewer studies have been performed in autochthonous tumours and none before and after systemic interventions. To perform interstitial fluid pressure measurements in normal tissues and autochthonous PDA, we used a Millar Mikro-Tip pressure catheter transducer connected to a control unit and data acquisition system (Ozerdem and Hargens, 2005; Provenzano *et al*, 2012). In normal tissues, IFP ranged from ∼8 mm Hg for the head of the pancreas, to 0.1 mm Hg for the liver, to −2.0 mm Hg in muscle, consistent with values measured in numerous other studies using a variety of methodologies (for examples, see Less *et al*, 1992 and Milosevic *et al*, 1998; reviewed in Aukland and Reed, 1993). These tissue IFPs are significantly below the typical intravascular pressures of the arterioles (40–80 mm Hg) and capillaries (15–40 mm Hg) that feed them, as originally hypothesised by Starling (1896). In contrast, IFPs in autochthonous PDAs were dramatically elevated, ranging from 75 to as high as 130 mm Hg, rivalling mean arterial pressures (Provenzano *et al*, 2012).

## Stromal hyaluronan in health and disease

HA is a large linear, negatively charged, and soluble macromolecule comprising *N*-acetyl glucosamine and glucuronic acid in alternating *β*-1,3 and *β*-1,4 linkages that figures prominently in the architecture, integrity, and malleability of tissues, particularly in dynamic processes such as embryogenesis and oncogenesis (Toole, 2004). HA also binds to the surface receptors, CD44 and RHAMM, activating signalling pathways that can promote cell survival, proliferation, adhesion, migration, and invasion. Its ability to imbibe and retain water contributes to the gel-like interstitia of many tissues and these same viscoelastic properties underlie the use of HA in a variety of cosmetic and clinical applications (Balazs and Denlinger, 1989). These properties together with the extraordinary abundance of HA beginning from the earliest stages of disease suggested a potentially critical role in establishing and defining the unique TME of PDA.

Homeostatic HA levels in mammalian tissues are maintained by the selective expression and activity of three different synthases, HAS1–3, and six different hyaluronidases (Toole, 2004). Originally identified as the ‘spreading factor’ from bovine testes (Chain and Duthie, 1939), purified hyaluronidase was later shown to represent a fragment of PH20, a GPI-anchored enzyme found on the acrosomal membrane of mammalian sperm that assists in fertilisation (Meyer *et al*, 1997). The purified testicular extract has been used historically to promote resorption of fluid and solutes administered subcutaneously. The unexpected palliation of disease in a multiple myeloma patient who received the extract for such an indication spurred investigations into the ability of the enzyme to enhance the efficacy of chemotherapies (Bruera *et al*, 1999). A number of cell autonomous and non-cell autonomous mechanisms have been proposed and continue to be studied (reviewed in Toole and Slomiany (2008) and Tredan *et al* (2007)).

## Preclinical trials: depleting HA reverses vascular collapse in primary and metastatic PDA and improves survival

The purified and recombinant forms of native hyaluronidase possess extremely short circulatory half-lives (*t*_1/2_<3 min) which, together with the development of hypersensitivity reactions, have precluded their use as systemic agents. Chemical conjugation of recombinant human PH20 with polyethylene glycol preserves enzymatic activity and extends the half-life to >10 h. Systemic administration of this agent can deplete HA from implanted tumour xenografts in immunocompromised mice and decrease IFP (Thompson *et al*, 2010). To directly address whether systemic administration of this enzyme could deplete HA from an autochthonous tumour and whether this would ameliorate the elevated IFP, we treated genetically engineered *Kras*^*LSL-G12D/+*^*;Trp53*^*LSL-R172H/+*^;*Cre* (*KPC*) mice with established invasive PDA with intravenous injections of PEGPH20. Significant intratumoral depletion of HA and reduction of IFP occurred within 2 h and peaked at 24 h (Provenzano *et al*, 2012). The reduction in IFP correspondingly resulted in a dramatic increase in patent tumour vessels without affecting overall vessel number, and ready penetration of chemotherapies across the tumour bed. Interestingly, although stromal HA is similarly depleted from a number of normal tissues in the animals, including heart, lungs, intestine, and liver, no overt untoward effects on organ function and overall health were observed.

When combined with the conventional cytotoxic, gemcitabine, in a randomised, placebo-controlled preclinical trial in *KPC* mice, PEGPH20 significantly increased objective response rate, decreased metastatic tumour burden, and prolonged median survival (Provenzano *et al*, 2012). Increased apoptosis was observed in both the pancreatic stellate cell and tumour epithelial cell compartments. After only a few weeks of combination therapy, the effects on HA levels and vascular perfusion persisted even if treatment ceased. Thus, the PDA stroma can be permanently remodelled in this manner, in principle permitting subsequent treatment with a wide variety of sequential and combination regimens. That metastatic tumour burden was decreased and not increased in the setting of increasing vascular access of cancer cells is also reassuring. A similar survival benefit with this combination therapy was independently observed (Jacobetz *et al*, 2013).

## Methods, models, and measurements of IFP

The fluid pressures we observed in autochthonous PDAs are significantly larger than those reported in a variety of *in vitro*, xenograft and allograft tumour model systems (reviewed in Heldin *et al*, 2004). Of course, an invasive carcinoma developing *in situ* in the native organ from spontaneously evolving precursor lesions is very different from the aforementioned model systems in several critical ways (see below). The apparatus used to measure fluid pressure has also varied. A number of systems have been used to measure fluid and tissue pressures over the last 50 years, including the needle (Scholander *et al*, 1968), modified wick-in-needle (Fadnes *et al*, 1977), micropipette (Wiederhielm, 1968), and implanted perforated capsule procedures (Floyer, 1966; Anas *et al*, 1968). Each of these systems has virtues and limitations, not the least of which is the need for sufficient skill to reproducibly fashion the custom glass needles used for the measurements (reviewed in Guyton *et al*, 1971). Indeed, the micropuncture glass capillary method is known to yield more reliable estimates of fluid pressures than the wick-in-needle because it produces less trauma and requires smaller volumes to record IFP (Heldin *et al*, 2004). The polyurethane ultraminiature pressure transducer used in our experiments was developed, in part, to overcome some of the limitations encountered in previous methodologies, and also to both simplify and increase the accuracy of the method (Zimmer and Millar, 1998). We also took a number of measures to ensure the fidelity of the measurements and that solid stresses (SSs) were not being measured in addition to fluid pressure. First, the instrument was calibrated to 0, 25 and 100 mm Hg before every experiment and confirmed at its conclusion. The catheter is also designed so that the sensor is situated in a side port a few mm from the end of the probe, and not at its very tip, to minimise any potential trauma or artifact during placement. In addition, we did not use the pressure catheter transducer directly to enter the tumour, but rather placed it in the track left behind after insertion and withdrawal of a 25-gauge needle. Third, for every measurement made, we ensured that the catheter could be freely and repeatedly inserted into and withdrawn from the preformed needle track several times without resistance and without any shearing of tissue (i.e., there was no adherence of tissue to the side-port sensor). Finally, we confirmed that IFP in the normal head of the pancreas dropped from a mean of 8 mm Hg to zero or even slightly negative pressure (−2.7 to 0.8 mm Hg) within minutes of the cessation of cardiac function and tissue blood flow, as expected. In normal pancreas tissue, the interstitial fluid space is in equilibrium with and directly chronicles mean vascular pressures (MVP). For all of these reasons, we concluded that the piezoelectric catheter transducer was not measuring SS. In fact, the theoretical possibility of solid pressure artifacts with this apparatus was specifically examined by Ozerdem and Hargens (2005) who found no evidence for such. Ozerdem (2009) went on later to describe the Millar SPC320 catheter as a ‘gold standard reference’.

**Figure 2:**
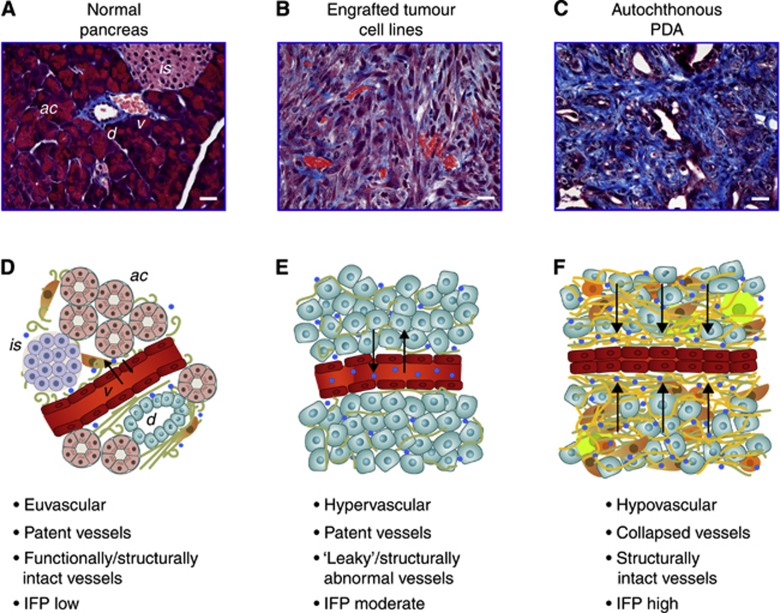
**Fluid and solid mechanics in distinct physiological states.** (**A**–**C**) Masson’s trichrome staining of normal pancreas, engrafted pancreas cancer cells, and autochthonous PDA. (**A**) Normal pancreata are composed largely of three epithelial compartments, namely ductal (*d*), acinar (*ac*), and islet cells (*is*), as well as ample functional vasculature and a scarce ECM. In tumours engrafted from purified carcinoma cells (**B**), the ECM is modest and numerous patent vessels are present. In contrast, autochthonous PDA (**C**) is dominated by a robust desmoplasia and a largely collapsed vasculature resulting in extremely limited perfusion. (**D**–**F**) Schema for distinct states (for illustrative purposes only and not drawn to scale). (**D**) In normal tissue, the interstitial fluid pressure is low and dependent upon the vascular pressure and the oncotic gradient. (**E**) Tumour implants of isolated cancer cells or lines possess a moderate IFP that is also related to vascular pressure. (**F**) In autochthonous PDA, the IFP is very high and the vasculature already collapsed. While the exact mechanisms of the elevated IFP remain to be elucidated, our results allow us to formulate a number of testable hypotheses for the genesis and maintenance of these pressures and mechanics. After vascular collapse, free and HA-bound fluid is trapped in the interstitial space (initially at pressures that result from communication with the vascular space before collapse, e.g., ∼20 to 50 mm Hg plus additional swelling pressures transmitted though the fluid during and shortly after collapse). Following vascular collapse, we further hypothesise that the pressure in this now compartmentalised fluid continues to increase, contributed to by a combination of events in the solid components of the tumour. First, solid stress continues to increase and acts on adjacent fluid through ongoing ECM production, tumour cell and fibroblast proliferation, and immune cell infiltration; as these components increase the density of the tumour, fluid pressure will correspondingly rise. Second, we propose that cells, activated by tumour-expanding pressures, resist this deformation by increasing cellular contractile force to actively compact the tumour, further elevating fluid pressure. As a consequence of these events, fluid pressure in PDA is extremely high. We suggest that digestion of HA by treatment with PEGPH20 liberates bound water and also relaxes the hydrogel being actively counterposed by mechanical forces. As pressures begin to drop, expanding vessels permit mobilisation of excess fluid into the circulation. The rush of free fluid and relaxation of physical constraints may also now permit some direct leaking of fluid out of the tumour. For (**D–F**), ductal epithelial/PDA cells are shown in aqua, stellate cells in brown, macrophages in green, and T cells in orange. Collagen is illustrated in green and HA in yellow. Arrows indicate fluid pressures and small blue circles indicate water molecules. Scale bars, 25 *μ*m for (**A–C**).

Our observations and conclusions differ from that derived from an extensive body of work employing novel and highly creative *in vitro* and tumour explant systems including ‘tissue-isolated tumours’ comprising an externalised ovarian fat pad injected with a rat mammary adenocarcinoma ‘tumour slurry’ (Sevick and Jain, 1989); ‘tumour spheroids’ comprising cell lines seeded into agarose gels (Helmlinger *et al*, 1997); and a variety of subcutaneous tumour cell line transplant systems (reviewed in Baxter and Jain, 1989). Shared properties among these experimental systems have allowed a sophisticated working model of tumour physiology to be proposed (Figure 2), characterised by: (1) hypervascularity; (2) markedly increased vascular permeability and hydraulic conductivity (i.e., ‘leaky’ vessels); and (3) ‘open’ systems in which the interstitial fluid space is in equilibrium with the intravascular space and fluid can ‘ooze out of the periphery’ (Boucher and Jain, 1992). As Jain and colleagues have elegantly demonstrated, elevated IFP in these contexts is due primarily to leaky vessels and dysfunctional lymphatics and can rise as high as microvascular pressure (MVP) (Jain, 1990). Moreover, because the intra- and extravascular fluid spaces are in equilibrium, changes in MVP are rapidly reflected in changes in IFP, which both plunge towards zero when the heart is stopped (Netti *et al*, 1995). The investigators concluded that IFP cannot collapse vessels and that, instead, vascular collapse is caused by SS from proliferating cancer cells (Boucher and Jain, 1992; Padera *et al*, 2004). From these observations, it seems reasonable to suggest that ‘collapsed lymphatic and blood vessels are known to contribute to elevated IFP and not the other way around’ (Stylianopoulos *et al*, 2012).

Unexpected challenges to this framework arise when confronting autochthonous PDAs. Properties of these tumours subvert the working assumptions derived from the aforementioned studies in many important ways (Figure 2). First, both human (Park *et al*, 2009) and murine PDAs (Olive *et al*, 2009; Provenzano *et al*, 2013) are profoundly hypovascular, in contradistinction to the systems described above. Second, the vasculature is characterised by widespread collapse. Third, the tumour vessels in PDA appear to be functionally and ultrastructurally intact (Jacobetz *et al*, 2013), have preserved interendothelial junctions, and are notably lacking in the fenestrae that characterise ‘leaky’ systems (Dvorak *et al*, 1988; Roberts and Palade, 1997). Further, despite the extremely elevated interstitial pressures, fluid is not ‘squeezed’ out from the tumour as would be expected if the system was in open communication with its environment, and small molecules are unable to effectively penetrate the tumour when administered either intravenously (Provenzano *et al*, 2012) or intraperitoneally (i.e., directly bathing the tumour) (Olive *et al*, 2009). Applying perhaps the most stringent criterion, intratumoral IFP does not drop after cessation of cardiac function in an animal with autochthonous PDA, even up to 40 min later, confirming that the intravascular and interstitial fluid spaces are largely disconnected (Provenzano *et al*, 2012). How PDA cells survive such a profoundly hostile microenvironment is worthy of study in its own right but likely includes a reliance on alternate metabolic pathways (Roberts and Borges, 1955; Warburg, 1956), transient microregional variations in pressures and blood flow (Heldin *et al*, 2004), and autophagy (Yang *et al*, 2011), among other possibilities. Nevertheless, to a first approximation, PDA would appear to behave as a ‘closed’ rather than open system, a reality that has profound implications for the generation and maintenance of elevated fluid pressures, as well as the relationship between IFP and SS. These considerations become especially germane when considering therapeutic strategies. Xenografts from both human (Hertel *et al*, 1990) and mouse respond readily to gemcitabine, in stark contrast to their respective autochthonous counterparts (Olive *et al*, 2009). Agents that inhibit or normalise angiogenesis are not effective in treating PDA in either humans (Kindler *et al*, 2010, 2011) or mice (Olson *et al*, 2011), despite their proven utility in typically hypervascular pancreatic neuroendocrine tumours (Raymond *et al*, 2011) and other malignancies (Jain, 2005). It is important to note that SS is, by definition, exerted only at points of direct contact, whereas a fluid pressure transmits force in all vectorial directions (Guyton *et al*, 1971). Thus, it is unlikely that a sufficiently large vascular surface area is in direct contact with solid tissue elements (primarily proliferating cells) and exerting a high enough pressure to collapse the majority of vessels in the tumour bed. Indeed, PDAs are paucicellular, particularly with regard to their epithelial cell content (Hruban, 2007), and the mean distances between tumour cell and vessel are relatively high (Olive *et al*, 2009). Thus, SS may contribute to vascular collapse in PDA, but IFP appears to predominate. In the end, it is likely that more than one working model of tumour physiology will be required to address and inform interventions across the spectrum of human solid malignancies.

Finally, how then to explain the profoundly elevated fluid pressures and vascular collapse in PDA? Do fluid pressures collapse vessels or do collapsed vessels lead to increased fluid pressures? The specific features of the stromal compartment and the intact ultrastructure of the vessels in autochthonous PDA provide the requisite properties to understand the phenomenon. The large extended HA polymers can imbibe significant amounts of water and organise it into a hydrogel, creating an immobilised fluid phase in equilibrium with freely mobile water (Aukland and Reed, 1993). The gel, nevertheless, also possesses an elastic modulus, a property of solids (Katchalsky, 1954). Thus, hydrated HA is viscoelastic and several types of pressure are associated with the HA hydrogel including: fluid pressure; a surface pressure; and a colloid osmotic pressure, contributed to by the Donnan equilibrium effect, and which itself can rise significantly and non-linearly in proportion to increasing HA concentration to become quite substantial (Tanford, 1961; Ogston, 1966; Guyton *et al*, 1971). Tethered collagen fibrillary and non-collagenous mirofibrillary structures with varying degrees of cross-linkage compound this complexity, given their ability to create microcompartments within the overall tumour architecture, while providing a scaffold for active cell contractility to constrain an expanding HA hydrogel (Figure 2). In open systems, SS and IFP can be considered additive to estimate a total tissue pressure (TTP). However, in the relatively closed system that more closely approximates an autochthonous PDA, the TTP is the IFP, which is transmitted through the solid matrix structure as well. Indeed, this is not unlike how hydraulic systems operate and generate force. Acute angle-closure glaucoma provides another apt example of the physiologic consequences of elevating fluid pressures in a confined space with free fluid and a hydrogel. Accumulation of aqueous humour (free fluid) in the posterior chamber of the eye acts in concert with the vitreous body – a hydrogel comprising HA, collagen, proteoglycans and both free and bound water – to impinge upon ocular blood flow and the optic nerve, threatening the eye.

The precise mechanics and interrelationships among the free fluid, solid, and viscoelastic phases in PDA aside, it is important not to lose sight of the most relevant finding from these studies: enzymatic degradation of HA through systemic administration of a clinical-grade reagent renders PDA more uniformly permeable to chemotherapeutics, revealing an unexpected and profound sensitivity to agents that have previously been shown to be ineffective. This finding permits the rational investigation of an entire array of small molecule, antibody, and cell-based therapies for PDA, in association with HA degradation. It has the potential, currently under active clinical investigation (NCT01453153), to influence the fundamental strategy in treating patients with this dreaded disease.

## Conclusions

The complex cellular, molecular, and mechanical features of the desmoplastic response in PDA presents challenges to effective treatment, as well potential vulnerabilities to exploit. In creating a drug-free sanctuary, the tumour also presumably remains sensitive, at least initially, to the chemotherapies it has been shielded from rather than selected to resist. Although intrinsic mechanisms of disease resistance will no doubt emerge, breaching the sanctuary with a systemically deliverable enzyme offers the prospect of unprecedented, sustained vascular access to the PDA primary tumour bed and metastases. This will enable not only rigorous evaluation of new treatment strategies, but also exploration of distinct sequencing and schedules of various treatment modalities in the attempt to remain one step ahead of the tumour. Indeed, re-examining the long list of clinically approved agents previously thought to be ineffective in PDA would be an excellent place to start. Additional mechanisms to reduce IFP should also be explored including targeting other ECM components (such as collagen, versican, or decorin) or inhibiting contractile forces generated by fibroblasts. Restoring physiologic blood flow, oxygen, and nutrients to a pancreas cancer will undoubtedly alter its metabolism, and these changes may present additional therapeutic targets and also new mechanisms of resistance. However, the idea that the complex and impervious pancreas cancer ‘organ’ can be perturbed dramatically is itself encouraging. It cannot be long before this literal and figurative entry into pancreas cancer pathophysiology results in a more effective therapeutic armamentarium.
